# Multidrug- and Extensively Drug-Resistant Uropathogenic *Escherichia coli* Clinical Strains: Phylogenetic Groups Widely Associated with Integrons Maintain High Genetic Diversity

**DOI:** 10.3389/fmicb.2016.02042

**Published:** 2016-12-21

**Authors:** Sara A. Ochoa, Ariadnna Cruz-Córdova, Victor M. Luna-Pineda, Juan P. Reyes-Grajeda, Vicenta Cázares-Domínguez, Gerardo Escalona, Ma. Eugenia Sepúlveda-González, Fernanda López-Montiel, José Arellano-Galindo, Briceida López-Martínez, Israel Parra-Ortega, Silvia Giono-Cerezo, Rigoberto Hernández-Castro, Daniela de la Rosa-Zamboni, Juan Xicohtencatl-Cortes

**Affiliations:** ^1^Laboratorio de Investigación en Bacteriología Intestinal, Hospital Infantil de México Federico GómezMexico City, Mexico; ^2^Posgrado en Ciencias Químico-Biológicas, Escuela Nacional de Ciencias Biológicas, Instituto Politécnico NacionalMexico City, Mexico; ^3^Laboratorio de Bacteriología Médica, Escuela Nacional de Ciencias Biológicas, Instituto Politécnico NacionalMexico City, Mexico; ^4^Laboratorio de Estructura de Proteínas, Instituto Nacional de Medicina GenómicaMexico City, Mexico; ^5^Área de Virología, Laboratorio de Infectología, Hospital Infantil de México Federico GómezMexico City, Mexico; ^6^Subdirección de Servicios Auxiliares de Diagnóstico, Hospital Infantil de México Federico GómezMexico City, Mexico; ^7^Laboratorio Clínico, Hospital Infantil de México Federico GómezMexico City, Mexico; ^8^Departamento de Ecología de Agentes Patógenos, Hospital General “Dr. Manuel Gea González,”Mexico City, Mexico; ^9^Epidemiología Hospitalaria, Hospital Infantil de México Federico GómezMexico City, Mexico

**Keywords:** UPEC, multidrug resistance, virulence genes, phylogenetic groups, PFGE

## Abstract

In recent years, an increase of uropathogenic *Escherichia coli* (UPEC) strains with Multidrug-resistant (MDR) and Extensively Drug-resistant (XDR) profiles that complicate therapy for urinary tract infections (UTIs) has been observed and has directly impacted costs and extended hospital stays. The aim of this study was to determine MDR- and XDR-UPEC clinical strains, their virulence genes, their phylogenetic groups and to ascertain their relationship with integrons and genetic diversity. From a collection of 500 UPEC strains, 103 were selected with MDR and XDR characteristics. MDR-UPEC strains were mainly associated with phylogenetic groups D (54.87%) and B2 (39.02%) with a high percentage (≥70%) of several fimbrial genes (*ecpA, fimH, csgA*, and *papG*II), an iron uptake gene (*chuA*), and a toxin gene (*hlyA*). In addition, a moderate frequency (40–70%) of other genes (*iutD, tosA*, and *bcs*A) was observed. XDR-UPEC strains were predominantly associated with phylogenetic groups B2 (47.61%) and D (42.85%), which grouped with ≥80 virulence genes, including *ecpA, fimH, csgA, papG*II, *iutD*, and *chuA*. A moderate frequency (40–70%) of the *tosA* and *hlyA* genes was observed. The class 1 and 2 integrons that were identified in the MDR- and XDR-UPEC strains were associated with phylogenetic groups D, B2, and A, while the XDR-UPEC strains that were associated with phylogenetic groups B2, D, and A showed an extended-spectrum beta-lactamase (ESBL) phenotype. The modifying enzymes (*aad*A1, *aad*B, *aac*C, *ant*1, *dfr*A1, *dfr*A17, and *aad*A4) that were identified in the variable region of class 1 and 2 integrons from the MDR strains showed resistance to gentamycin (56.25 and 66.66%, respectively) and trimethoprim-sulfamethoxazole (84.61 and 66.66%, respectively). The MDR- and XDR-UPEC strains were distributed into seven clusters and were closely related to phylogenic groups B2 and D. The diversity analysis by PFGE showed 42.68% of clones of MDR-UPEC and no clonal association in the XDR-UPEC strains. In conclusion, phylogenetic groups including virulence genes are widely associated with two integron classes (1 and 2) in MDR- and XDR-UPEC strains.

## Introduction

Urinary tract infections (UTIs) are one of the most common bacterial infectious diseases in humans, with ~150 million cases estimated annually worldwide (Flores-Mireles et al., 2015). Uropathogenic *Escherichia coli* (UPEC) causes 80–90% of community-acquired UTIs and 40–50% of nosocomial-acquired UTIs (Foxman, 2010; Foxman et al., 2012; Toval et al., 2014; Flores-Mireles et al., 2015). UTIs associated with UPEC usually begin as bladder infections (cystitis) but can develop into acute kidney infections (pyelonephritis) and even infections of the bloodstream (urosepsis) (Flores-Mireles et al., 2015). UPEC pathogenesis involves several virulence factors to resist urine flow, to trigger host-bacterial cell signaling pathways, and to establish infection (Siliano et al., 2010; Jadhav et al., 2011; Alteri and Mobley, 2012). FimH (Type 1 fimbriae), EcpA (*E. coli* Common Pilus), CsgA protein (curli), PapGI, PapGII, and PapGIII variants (P fimbriae) are fimbrial adhesins that participate in UPEC adherence and colonize the bladder epithelium (Mulvey et al., 1998; Lane and Mobley, 2007; Cegelski et al., 2009; Saldaña et al., 2014). Iron uptake proteins (aerobactin, IutD), toxin protein (α-hemolysin, HlyA), type 1 secretion A (TosA), and surface glycan proteins (cellulose and BcsA) participate in UPEC pathogenesis (Gao et al., 2012; Kudinha et al., 2013; Engstrom et al., 2014; Lüthje and Brauner, 2014; Subashchandrabose and Mobley, 2015). UPEC clinical strains are associated with four main phylogenetic groups (A, B1, B2, and D) that are characterized by the existence of genetic markers, such as *chuA, yjaA*, and the DNA fragment TspE4.C2 (Lee et al., 2016). Phylogenetic groups B2 and D are commonly related to UPEC strains collected from patients with UTIs; however, at a lower frequency, these strains are also related to phylogenetic groups A and B1. In addition, multidrug-resistance (MDR) and virulence factors described in UPEC strains are associated with phylogenetic group B2 (Derakhshandeh et al., 2015; Ferjani et al., 2015). Recently, the MDR-phenotype was found to be related to phylogenetic groups A and D; although, UPEC strains susceptible to most antibiotics are also related to phylogenetic group B2 (Ejrnæs et al., 2011).

The mechanisms that contribute to resistance in UPEC strains are as follows: (1) inactivation of hydrolytic enzymes by β-lactamases; (2) no hydrolytic enzyme inactivation by aminoglycoside acetyl transferase enzymes; (3) permeability alteration through active efflux pumps; (4) inactivation of the target site; and (5) resistance acquired by the horizontal transfer of genetic elements, such as insertion sequences, gene cassettes, integrons, and transposons (Peleg and Hooper, 2010). Integrons generally constitute an integrase gene (*intI*), an attachment site (*attI*), and a promoter (Pc) that induces the expression of any suitable integrated gene(s). Additionally, two classes of integrons (classes 1 and 2) have been identified and characterized in MDR-UPEC clinical strains (Stalder et al., 2012; Deng et al., 2015). These integrons carry several genes that encode proteins that participate in antibiotic resistance. These integrons are frequently embedded in mobile DNA elements, such as transposons and conjugative plasmids, to spread horizontally throughout bacterial populations (Stalder et al., 2012; Deng et al., 2015). However, the integrons in MDR-UPEC clinical strains and their relationship with virulence factors and clonality have been poorly studied. In recent years, an increase in UPEC strains with an extensively drug-resistant (XDR) profile complicating UTI therapy has been observed (Dehbanipour et al., 2016; Hadifar et al., 2016). The aim of this study was to identify MDR- and XDR-UPEC clinical strains and their virulence genes and phylogenetic groups and to ascertain their relationship with integrons and their genetic diversity.

## Materials and methods

### Bacterial strains

A total of 500 UPEC clinical strains were collected from 2010 to 2012 at the Hospital Infantil de México Federico Gómez (HIMFG) from pediatric patients with community- and nosocomial-acquired UTIs. The UPEC strains were selected considering an average bacterial count of ≥100,000 CFU/mL as determined by Kass-Sandford (Kass, 1957). UPEC strains associated with persistent UTIs were isolated from cultures from the same patient taken in different months. UPEC strains were cultured in trypticase soy broth (TSA), MacConkey medium (Mac), Luria-Bertani medium (LB), and Mueller-Hinton Broth (MHB) (Difco-Becton Dickinson, NJ, USA) at 37°C for 24 h according to assay type. Additionally, UPEC strains were stored in cryovials at −70°C with Brain Heart Infusion broth (BHI; Difco-Becton, Dickinson, NJ, USA) containing 25% glycerol (Sigma-Aldrich, St. Louis, MO, USA). Only one isolate from each infectious episode was included in the study, and isolates were considered to come from the same episode if they were collected <30 days apart. Thus, all MDR- and XDR-UPEC strains were isolated from 103 infectious episodes in 71 patients, and 8 patients experienced more than one episode.

### Antimicrobial susceptibility and phenotypic detection of ESBLs

The minimum inhibitory concentration (MIC) as determined by the MHB microdilution method was used to evaluate the antimicrobial susceptibility of 500 UPEC clinical strains according to the guidelines of the Clinical and Laboratory Standards Institute (CLSI, 2016). MDR strains were defined as having acquired no susceptibility to at least one antibiotic in three or more classes. XDR strains were defined as having non-susceptibility to at least one agent in all but two or fewer antibiotic classes (Magiorakos et al., 2012). The MIC for each antibiotic was compared to the standard values of the CLSI. The antibiotic panel that was used included ampicillin (AM; Sigma-Aldrich, St. Louis, MO, USA), amoxicillin-clavulanate (AMC; Great West Road, Brentford Middlesex, UK), ticarcillin-clavulanate (TIM; Gold Biotechnology, Inc., Ashby Road, St. Louis, MO), piperacillin-tazobactam (TZP; Siemens Medical Solutions USA, Inc., Valley Stream Parkway, Malvern, PA, USA), cephalothin (CF; Eli Lilly and Company, S Harding St, Indianapolis, IN, USA), cefaclor (CEC; Phadia Laboratory Systems, Thermo Scientific, Wyman Street, Waltham, MA, USA), ceftazidime (CAZ; Roselle Rd, Schaumburg, IL, USA), aztreonam (ATM; Bristol-Myers Squibb Corporate, Park Avenue, NY, USA), norfloxacin (NOR), ofloxacin (OFX; MP Biomedicals, Solon, OH, USA), meropenem (MEM), imipenem (IPM; AstraZeneca Pharmaceuticals LP, Wilmington, DE, USA), gentamycin (GM; Schering-Plough Pharmaceuticals, Kenilworth, NJ, USA), ceftriaxone (CRO), trimethoprim-sulfamethoxazole (SXT; Roche, Basel, Switzerland), tetracycline (TE; Heritage Pharmaceuticals Inc., Fieldcrest Avenue, Edison, NJ, USA), and nitrofurantoin (F/M; McKesson Pharmaceutical, One Post Street, San Francisco, CA, USA). *E. coli* ATCC 25922 and *Pseudomonas aeruginosa* ATCC 27853 were used as controls.

The extended-spectrum beta-lactamases (ESBLs) were phenotypically detected as previously recommended by CLSI using the double-disc synergy test based on the synergistic effect between clavulanic acid (inhibitor of ESBLs) and β-lactam antibiotics (cefotaxime, CRO, CAZ, cefepime, cefpirome, and ATM). Additionally, ESBLs were detected using an individual disk that was tested with/without clavulanic acid (10 μg/mL) and by the Hodge test using *Klebsiella pneumoniae* ATCC 700603 (ESBL+) and *E. coli* ATCC 25922 (ESBL-) as control strains (CLSI, 2016).

### Phylogenetic groups

DNA was extracted from the MDR- and XDR-UPEC clinical strains cultured in LB using the Wizard® Genomic DNA Purification Kit (Promega Corporation, Woods Hollow Road, Madison, WI, USA) according to the manufacturer's instructions. Multiplex polymerase chain reaction (PCR) assays were used to determine the presence of *chuA, yjaA*, and one genomic fragment (TspE4.C2) (Clermont et al., 2000) in MDR- and XDR-UPEC clinical strains (Table S1). The PCR products were resolved by electrophoresis on a 1.5% agarose gel (Promega Corporation, Woods Hollow Road, Madison, WI, USA) that was stained with ethidium bromide solution (Sigma-Aldrich, St. Louis, MO, USA) and visualized using a Gel Imaging System (ChemiDoc MP System, Bio-Rad; Mexico). According to the amplicon data, the strains were assigned to phylogenetic groups as follows: group B2 (*chuA*^+^/*yjaA*^+^), group D (*chuA*^+^/*yjaA*^−^), group B1 (*chuA*^−^/TspE4.C2^+^), and group A (*chuA*^−^/TspE4.C2^−^) (Clermont et al., 2000).

### Identification of virulence genes

Eight virulence-associated genes [three variants (I, II, and III) of *papG* (PapG), *fimH* (FimH), *bcsA* (cellulose), *csgA* (CsgA), *ecpA* (EcpA), *iutD* (aerobactin), *hlyA* (α-hemolysin), and *tosA* (type 1 secretion A)] from MDR- and XDR-UPEC clinical strains were identified by multiplex PCR using specific primers (Table S1). CFT073 *E. coli* was used as a positive control.

### Identification of class 1, 2, and 3 integrase genes

Integrons in the MDR- and XDR-UPEC strains were detected by multiplex PCR, which amplified the conserved region of the integrase-encoded genes *intI*1, *intI*2, and *intI*3 using specific primers (Table S1). MDR-UPEC strains 502U1-0412 and 674U-0612 were used as positive controls for class 1 and 2 integrons, respectively.

### Sequencing of the amplified integron gene cassettes

The variable regions of class 1 and 2 integrons from the MDR-UPEC clinical strains were amplified by PCR using a high-fidelity *Pfu* polymerase of Thermo-Fisher Scientific (CA, USA) (Table S1). The amplicons were cleaned and concentrated using the Zymo DNA Clean and Concentrator of Zymo Research (CA, USA) and subjected to next-generation sequencing on a NexSeq500 System (Illumina, CA, USA), which was performed at “Unidad de Secuenciación del Instituto Nacional de Medicina Genómica” (CDMX, Mexico). The sequences were analyzed and assembled using ClustalO, ORF Finder (Open Reading Frame Finder), and BLAST (Basic Local Alignment Search Tool) from the NCBI (National Center of Biotechnology Information) (Sievers et al., 2011; Soleimani et al., 2014).

### PFGE analysis in MDR- and XDR-UPEC strains

A phylogenetic analysis of MDR- and XDR-UPEC clinical strains was performed using pulsed-field gel electrophoresis (PFGE) following the specific modifications of the protocols established by the “Laboratorio de Investigación en Bacteriología Intestinal, HIMFG” (Ochoa et al., 2015). Briefly, the samples were digested with 2 U of *Xba*l (Invitrogen, Life Technologies, Wyman Street, Waltham, MA, USA) at 37°C for 4 h and separated by electrophoresis in a 1.2% ultrapure DNA agarose gel (Bio-Rad, Life Science Research, Hercules, CA, USA) that had been previously dissolved in 0.5X TBE. The samples were run on a CHEF Mapper system (Bio-Rad Life Science Research, Hercules, CA, USA) for 23 h at 200 V (6 V/cm) under two different linear ramped pulse times: 1–10 s and 10–40 s. The gels containing the macrorestriction products were stained for 40 min with 0.5 mg/mL ethidium bromide (Sigma-Aldrich St. Louis, MO, USA) and visualized under UV light using an Imaging System (ChemiDoc MP System®, Bio-Rad, Mexico City, Mexico). A lambda ladder PFGE marker (New England Biolabs, Hertfordshire, England, UK) was used as a molecular weight marker. PFGE patterns were analyzed using NTSYS-pc software (version 2.0, Applied Biostatistics, Inc., NY, USA) with the Sørensen-Dice similarity coefficient and a cluster analysis of the Unweighted Pair Group Method using Arithmetic averages (UPGMA) (Dice, 1945). The clonality of the UPEC strains was evaluated considering the Criteria of Tenover (Tenover et al., 1995).

### Statistical analysis

Fisher's exact test and Fisher's PLSD ANOVA were used to identify significant differences in the data at *p* < 0.05.

## Results

### MDR and XDR profiles in the UPEC strains

According to the resistance profile to 10 classes of antibiotics, 16.40% (82/500) of the UPEC strains were MDR, and 4.20% (21/500) were XDR. Additionally, MDR- and XDR-UPEC strains were isolated from hospitalized patients with complicated UTIs from different wards of HIMFG, such as Nephrology, Urology, PICU (Pediatric Intensive Care Unit), NICU (Neonatal Intensive Care Unit), Oncology, and Pediatrics. From these, 82 MDR-UPEC strains were distributed as follows: 31.70% (26/82) were resistant to three classes of antibiotics, 28.04% (23/82) to four classes, 24.39% (20/82) to five classes, 9.75% (8/82) to six classes, and 6.09% (5/82) to seven classes. Furthermore, in eight patients, 40 MDR-UPEC strains were collected with five isolates from each patient. Moreover, 21 of the XDR-UPEC strains were distributed as follows: 52.38% (11/21) of were resistant to eight classes of antibiotics and 47.61% (10/21) to nine classes (Tables S2, S3). Interestingly, 76.19% (16/21) of XDR-UPEC strains had an ESBL phenotype (*p* ≤ 0.028) compared to 18.29% (15/82) of MDR-UPEC strains. Class 1 and 2 integrons were identified in 43.90% (36/82) and 2.43% (2/82) of MDR-UPEC strains, respectively. Additionally, 47.61% (10/21) of XDR-UPEC strains presented class 1 integrons, and 9.52% (2/21) presented class 2 integrons. These UPEC strains did not present class 3 integrons.

### MDR- and XDR-UPEC strains, phylogenetic groups, and their association with virulence genes

MDR-UPEC strains were distributed into three phylogenetic groups: 54.87% (45/82) to D, 39.02% (32/82) to B2, and 6.09% (5/82) to A; the XDR-UPEC strains were distributed as follows: 47.61% (10/21) to B2, 42.85% (9/21) to D, and 9.52% (2/21) to A. Moreover, phylogenetic group B1 was not identified in MDR- or XDR-UPEC strains. On the other hand, 28.19% (9/32), 11.12% (5/32) and 20% (1/32) of MDR-UPEC strains in groups B2, D, and A, respectively, were associated with the ESBL phenotype. In contrast, 80% (8/10), 66.67% (6/9), and 100% (2/2) of XDR-UPEC strains in groups B2, D, and A, respectively, produced ESBLs.

The *tosA* gene encoding the type 1 secretion A toxin was distributed at different percentages to phylogenetic groups A, B2, and D of MDR- and XDR-UPEC strains (Table 1). The *tosA* gene from MDR-UPEC strains associated with phylogenetic group B2 was significantly different (*p* ≤ 0.0001) from that from XDR-UPEC strains (Table 1). Fimbrial (*ecpA, fimH, csgA*, and *papG*II), iron uptake (*chuA, iutD*) and α-Hemolysin (*hlyA*) genes from MDR- and XDR-UPEC strains were highly distributed into phylogenetic groups B2 and D (Table 1). Additionally, the *papG*III variant gene of the MDR-UPEC strains was only distributed into phylogenetic group B2 and D, while the *papG*I variant gene was not identified in any UPEC strain (Table 1). The *bcs*A gene was distributed into phylogenetic groups as follows: 51.10% (23/45) to D, 43.75% (14/32) to B2 and 40% (2/5) to A of MDR-UPEC strains; however, XDR-UPEC strains carrying the *bcs*A gene were only distributed into phylogenetic group B2 (Table 1).

**Table 1:** **Phylogenetic groups associated with virulence genes in MDR- and XDR-UPEC clinical strains**.

**Variable**	**MDR-UPEC clinical strains**				**Total % (*n* = 82)**	**XDR-UPEC clinical strains**				**Total % (*n* = 21)**
	**Percentage in the phylogenetic groups**					**Percentage in the phylogenetic groups**				
	**A% (*n* = 5)**	**B1% (*n* = 0)**	**B2% (*n* = 32)**	**D% (*n* = 45)**		**A% (*n* = 2)**	**B1% (*n* = 0)**	**B2% (*n* = 10)**	**D% (*n* = 9)**	
**SURFACE GLYCAN^a^**										
*bcsA*	40 (2)	0 (0)	43.75 (14)	51.10 (23)	47.56 (39)	0 (0)	0 (0)	20 (2)	0 (0)	9.52 (2)
**FIMBRIAE/ADHESIN^b^**										
*ecpA*	100 (5)	0 (0)	96.87 (31)	95.55 (43)	96.34 (79)	100 (2)	0 (0)	100 (10)	100 (9)	100 (21)
*fimH*	100 (5)	0 (0)	100 (32)	97.76 (44)	98.78 (81)	0 (0)	0 (0)	90 (9)	88.89 (8)	80.95 (17)
*csgA*	80 (4)	0 (0)	96.87 (31)	97.76 (44)	96.34 (79)	100 (2)	0 (0)	100 (10)	100 (9)	100 (21)
*papG*I	0 (0)	0 (0)	0 (0)	0 (0)	0 (0)	0 (0)	0 (0)	0 (0)	0 (0)	0 (0)
*papG*II	40 (2)	0 (0)	90.62 (29)	97.76 (44)	91.46 (75)	50 (1)	0 (0)	100 (10)	100 (9)	95.23 (20)
*papG*III	0 (0)	0 (0)	15.62 (5)	2.22 (1)	7.31 (6)	0 (0)	0 (0)	0 (0)	0 (0)	0 (0)
**IRON UPTAKE^c^**										
*iutD*	60 (3)	0 (0)	65.62 (21)	51.10 (23)	57.31 (47)	50 (1)	0 (0)	100 (10)	77.76 (7)	85.71 (18)
*chuA*	0 (0)	0 (0)	100 (32)	100 (45)	93.90 (77)	0 (0)	0 (0)	100 (10)	100 (9)	90.47 (19)
**TOXINS^d^**										
*hlyA*	100 (5)	0 (0)	75 (24)	73.32 (33)	75.60 (62)	50 (1)	0 (0)	40 (4)	44.43 (4)	42.85 (9)
*tosA*	40 (2)	0 (0)	62.50 (20)	15.54 (7)	35.36 (29)	50 (1)	0 (0)	30 (3)	55.56 (5)	42.85 (9)

a *Cellulose (bcsA)*.

b *E. coli Common Pilus (ecpA), type 1 fimbriae (fimH), curli fimbriae (csgA), P fimbriae, PapG variant I (papGI), PapG variant II (papGII), PapG variant III (papGIII)*.

c *Aerobactin (iutD), E. coli haem-utilization-gene (chuA)*.

d *α-hemolysin (hlyA), type 1 secretion A (tosA)*.

### Integrons in MDR- and XDR-UPEC strains and their association with phylogenetic groups

MDR-UPEC strains with class 1 integrons were related to phylogenetic groups A, B2, and D [40% (2/5), 43.75% (14/32), and 44.43% (20/45), respectively]; under these same conditions, class 2 integrons were related to phylogenetic groups B2 and D [3.12% (1/32) and 2.22% (1/45), respectively] (Table 2). In the XDR-UPEC strains, class 1 integrons were related to phylogenetic groups B2, D, and A [30% (3/10), 55.56% (5/9), and 100% (2/2), respectively]; in addition, the class 2 integrons were associated with phylogenetic groups B2 and A in 10% (1/10) and 50% (1/2), respectively (Table 2).

**Table 2:** **Phylogenetic groups associated with integron classes and the ESBL phenotype in MDR- and XDR-UPEC clinical strains**.

**Resistance Mechanisms**	**MDR-UPEC clinical strains**				**Total % (*n* = 82)**	**XDR-UPEC clinical strains**				**Total % (*n* = 21)**
	**Percentage in the phylogenetic groups**					**Percentage in the phylogenetic groups**				
	**A% (*n* = 5)**	**B1% (*n* = 0)**	**B2% (*n* = 32)**	**D% (*n* = 45)**		**A% (*n* = 2)**	**B1% (*n* = 0)**	**B2% (*n* = 10)**	**D% (*n* = 9)**	
*intl*1	40 (2)	0 (0)	43.75 (14)	44.43 (20)	43.90 (36)	100 (2)	0 (0)	30 (3)	55.56 (5)	47.61 (10)
*intl*2	0 (0)	0 (0)	3.12 (1)	2.22 (1)	2.43 (2)	50 (1)	0 (0)	10 (1)	0 (0)	9.52 (2)
*intl*3	0 (0)	0 (0)	0 (0)	0 (0)	0 (0)	0 (0)	0 (0)	0 (0)	0 (0)	0 (0)
ESBLs	20 (1)	0 (0)	28.12 (9)	11.12 (5)	18.29 (15)	100 (2)	0 (0)	80 (8)	66.67 (6)	76.19 (16)

*Class 1 integrase (intl1), class 2 integrase (intl2), class 3 integrase (intl3), and extended-spectrum beta-lactamases (ESBLs)*.

### Analysis of class 1 and 2 integron sequences from MDR- and XDR-UPEC strains

A total of 56.52% (26/46) of MDR- and XDR-UPEC strains amplified the variable region of class 1 integrons with a product of ~1800 bp; in addition, 75% (3/4) of the MDR- and XDR-UPEC strains amplified the variable region of class 2 integrons with a product of ~2000 bp (Table 3). The sequencing analysis of ~1800-bp products showed the gene cassettes for *aad*A1 (aminoglycoside-3′-adenylyltransferase), *aad*B (aminoglucoside-2′-adenylyl-transferase), *aac*C (gentamicin-acetyl-transferase), *ant*1 (streptomycin-3′-adenylyltransferase), *dfr*A1 (dihydrofolate reductase type 1), and *dfr*A17 (streptomycin-3′-O-adenylyltransferase); their distribution is shown in Table 3. In contrast, the *aad*A1, *dfr*A1, and *aad*A4 (streptomycin-3′-adenylyl transferase) genes were identified in products of ~2000 bp (Table 3). Finally *aad*A1, *aad*B, *aac*C, *ant*1, *dfr*A1, and *dfr*A17, which encode modifying enzymes within the class 1 and 2 integrons from MDR- and XDR-UPEC strains, were associated with resistance to GM or SXT.

**Table 3:** **Sequencing of class 1 and 2 integrons and their association with gentamicin and trimethoprim-sulfamethoxazole**.

**Clinical strains**	**Class of Integron**	**Resistance profile**	**Phylogenetic groups**	**Size of gene cassettes (bp)**	**Identified gene cassettes**	**Susceptibility to GM μg/mL (R/S)**	**Susceptibility to SXT μg/mL (R/S)**
118U2	1	MDR (4)	D	~1800	*-dfr*A1 (Dihydrofolate reductase type 1)	8 (R)	>256 (R)
118U4	1	MDR (4)	D			8 (R)	>256 (R)
118U5	1	MDR (4)	D			8 (R)	>256 (R)
11U-0912	1	XDR (8)	B2			64 (R)	>256 (R)
433U1-0512	1	MDR (4)	D			0.5 (S)	>256 (R)
494U2-0412	1	MDR (5)	D			>256 (R)	0.5 (S)
502U-0412	1	MDR (4)	D			8 (R)	>256 (R)
54U-0612	1	MDR (3)	B2			16 (R)	0.25 (S)
647U-0712	1	MDR (5)	D			>256 (R)	>256 (R)
909U-0612	1	MDR (5)	D			2(S)	0.25 (S)
188U-1112	1	MDR (6)	D	~1800	-*aad*A1 (Aminoglycoside-3′-adenylyltransferase) -*dfr*A1 (Dihydrofolate reductase type 1)	32 (R)	>256 (R)
440U1	1	MDR (4)	B2			2 (S)	>256 (R)
440U2	1	MDR (3)	B2			2 (S)	>256 (R)
440U3	1	MDR (3)	B2			2 (S)	>256 (R)
440U4	1	MDR (3)	B2			2 (S)	>256 (R)
440U5	1	MDR (3)	B2			2 (S)	>256 (R)
945U2-0412	1	XDR (9)	D			>256 (R)	4 (R)
118U3	1	MDR (4)	D	~1800	-*aad*B (Aminoglucoside-2′-adenylyltransferase) -*dfr*A1 (Dihydrofolate reductase type 1)	8 (R)	>256 (R)
179U-1012	1	MDR (5)	D			32 (R)	>256 (R)
513U-0912	1	XDR (8)	D			>256 (R)	>256 (R)
720U-0712	1	XDR (8)	B2			8 (R)	4 (R)
86U-0612	1	MDR (3)	D			>256 (R)	>256 (R)
877U-1112	1	XDR (8)	D			>256 (R)	1 (S)
117U1-0512	1	MDR (5)	D	~1800	-*aac*C (Gentamicin-acetyltransferase -*dfr*A1 (Dihydrofolate reductase type 1) -*dfr*A17 (Streptomycin-3′-O-adenylyltransferase) -*ant*1 (Streptomycin-3′-adenylyltransferase)	0.5 (S)	>256 (R)
118U1	1	MDR (4)	D			8 (R)	>256 (R)
553U-1112	1	MDR (3)	D			0.5 (S)	>256 (R)
11U-0912	2	XDR (8)	B2	~2000	-*aad*A1 (Aminoglycoside-3′-adenylyltransferase) -*dfr*A1 (Dihydrofolate reductase type 1) -*aad*A4 (Streptomycin-3′-adenylyltransferase)	64 (R)	>256 (R)
188U-1112	2	MDR (6)	D			32 (R)	>256 (R)
674U-0612	2	MDR (4)	B2			4 (S)	2 (S)

*GM, Gentamicin; SXT, Trimethoprim-sulfamethoxazole; S, Sensitive; R, Resistant*.

### Genetic diversity of the MDR- and XDR-UPEC strains

**Figure 1:**
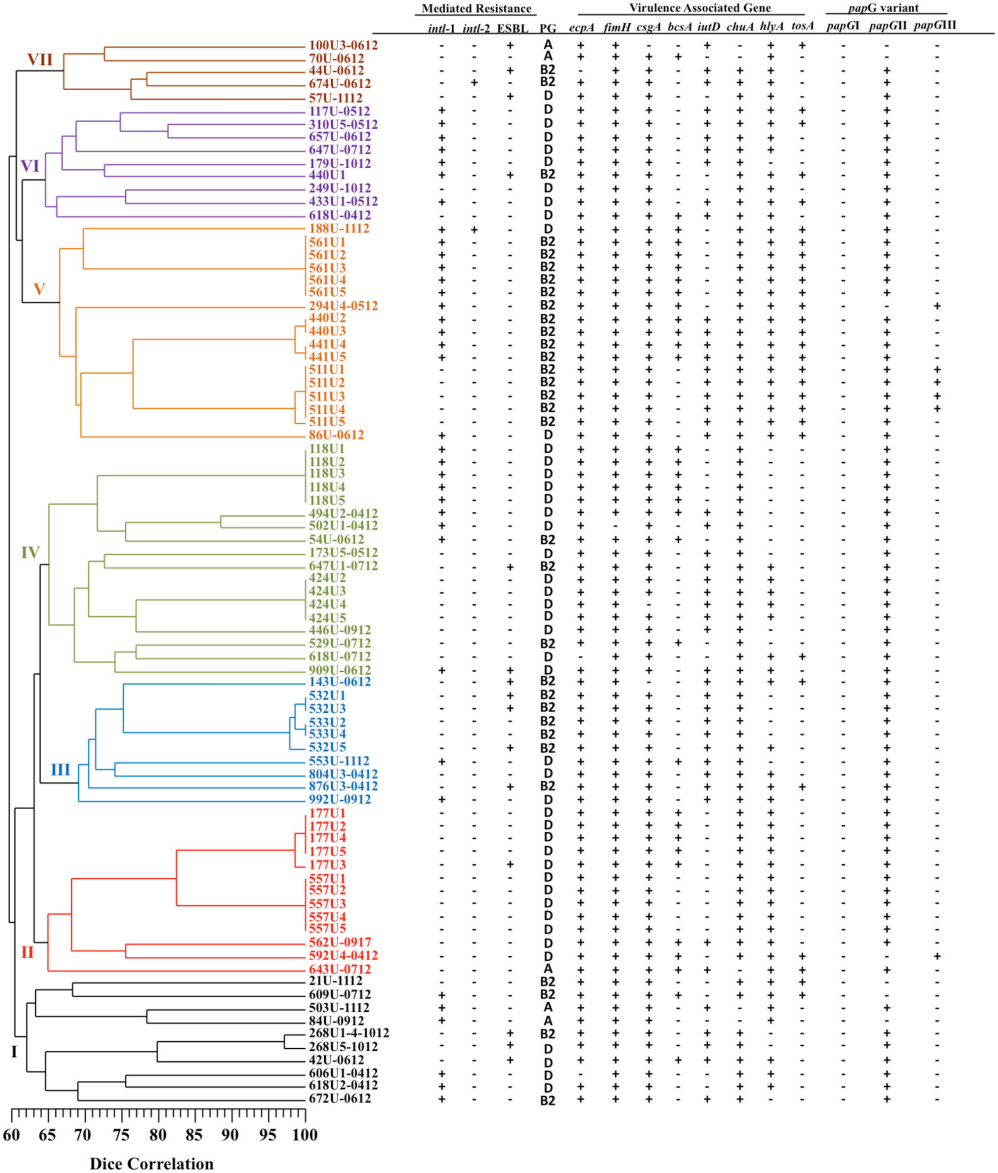
**PFGE analysis of 82 MDR-UPEC strains associated with virulence genes and their mechanisms of resistance**. A diversity analysis was performed using the Sørensen-Dice similarity coefficient in association with the UPGMA algorithm. Additionally, the dendrogram was evaluated using the cophenetic correlation coefficient obtained by the Mantel test, which indicated the dispersion of the data and showed a value of *r* = 0.8124. The seven clusters identified by PFGE are shown in different colors.

**Figure 2:**
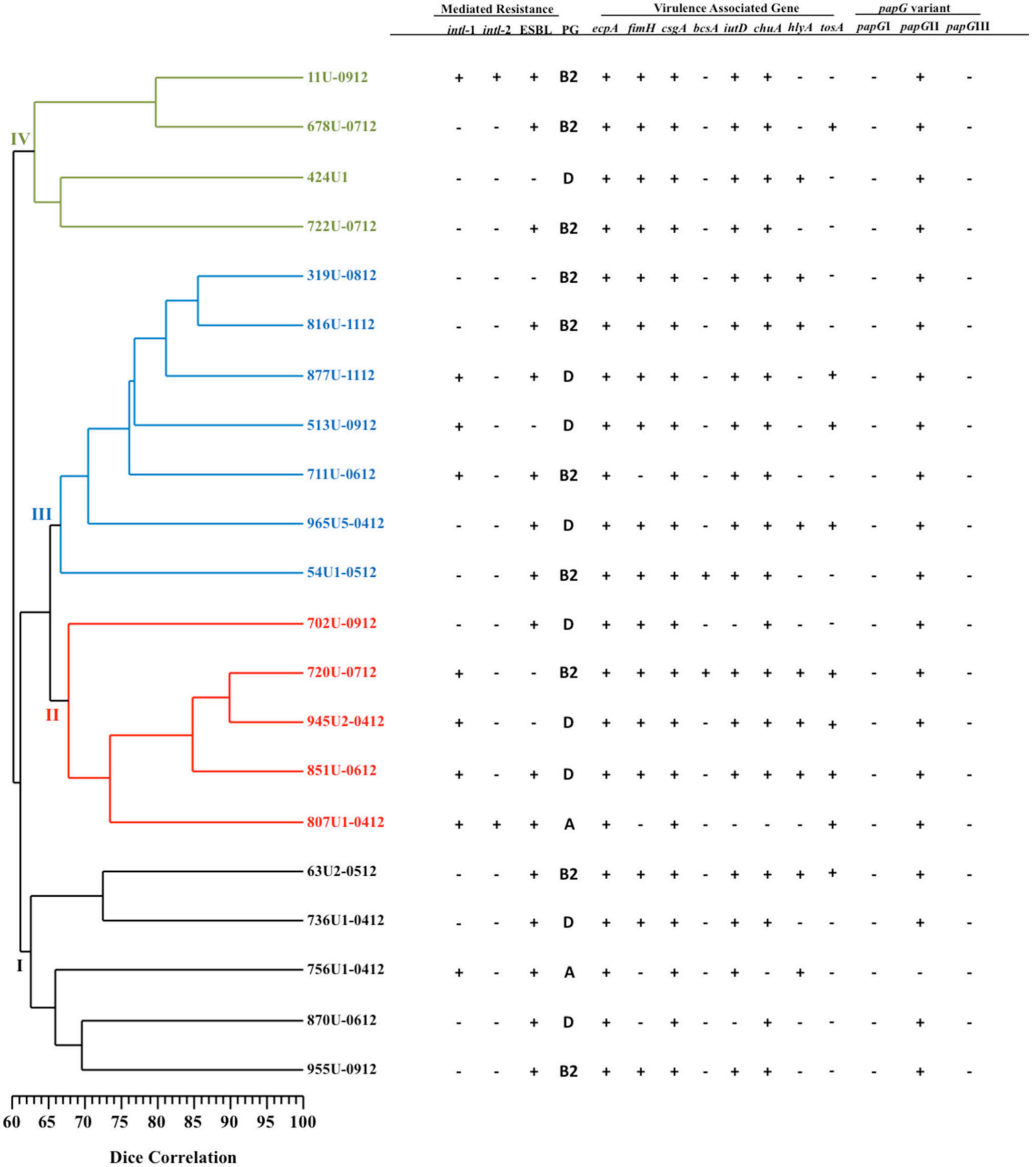
**PFGE analysis of 21 XDR-UPEC strains associated with virulence genes and their mechanisms of resistance**. The diversity analysis was performed using the Sørensen-Dice similarity coefficient in association with the UPGMA algorithm. Additionally, the dendrogram was evaluated using the cophenetic correlation coefficient obtained by the Mantel test, which indicated the dispersion of the data and showed a value of *r* = 0.8037. The four clusters identified by PFGE are shown in different colors.

Patterns consisting of 11–21 DNA fragments ranging in size from 48.5 to 436.5 Kb obtained from the PFGE assay of the MDR- (51 pulsotypes) and XDR- (21 pulsotypes) UPEC strains were used to construct two dendrograms with cophenetic correlation coefficients of 0.8124 and 0.8037 (Figures 1, 2). Furthermore, 12.19% (10/82) of the MDR-UPEC strains were distributed into cluster I, 15.85% (13/82) into cluster II, 12.19% (10/82) into cluster III, 21.95% (18/82) into cluster IV, 20.73% (17/82) into cluster V, 10.97% (9/82) into cluster VI, and 5% (5/82) into cluster VII (Figure 1). Of the seven clusters from the dendrogram, four clusters (II, III, IV, and V) grouped MDR-UPEC strains within isolates that were clonally related, collected at different times, and considered persistent; however, in clusters I, VI, and VII, a clonal relationship was not identified. Finally, XDR-UPEC strains were distributed into four clusters: 23.80% (5/21) into cluster I, 23.80% (5/21) into cluster II, 33.33% (7/21) into cluster III, and 19.04% (4/21) into cluster IV (Figure 2).

### Clonality, phylogenetic groups, virulence gene, and integrons in the MDR- and XDR-UPEC strains

The four clusters (II, III, IV, and V) grouping MDR-UPEC strains shared the presence of fimbrial genes (*ecpA, fimH, csgA*, and *papG*II) and an iron uptake gene (*chuA*). In clusters I, VI, and VII, a relationship between phylogenetic groups, integrons and virulence genes was not identified. In cluster II, two clonal groups associated with phylogenetic group D were identified: 557U (1–5 clones) characterized by the absence of the *bcsA* gene and 177U (1, 2, 4, and 5 clones) by the presence of the *bcsA* gene. In cluster III, two clonal groups associated with phylogenetic group B2 were identified: 532U (1 and 3 clones) characterized by the presence of the ESBL phenotype and 533U (2 and 4 clones) by the lack of the ESBL phenotype. In cluster IV, two clonal groups associated with phylogenetic group D were identified: 424U (2–5 clones) characterized by the presence of the *iutD* and *hlyA* genes and 118U (1–5 clones) by the presence of class 1 integrons and the *bcsA* gene. In addition, 424U4 lacked the *csgA* gene compared with 424U (2, 3, and 5 clones). In cluster V, four clonal groups [561U (1–5 clones), 440U (2 and 3 clones), 441U (4 and 5 clones), and 511U (1–5 clones)] associated with phylogenetic group B2 and with the *tosA* and *hlyA* genes were identified. Class 1 integrons and the *bcsA* gene were found in three clonal groups (561U, 440U, and 441U) and the *iutD* gene in three clonal groups (440U, 441U, and 511U). Interestingly, the *papG*III gene was only found in clonal group 511U associated with the *tosA* and *hlyA* genes (Figure 1).

## Discussion

Uncomplicated UTIs are commonly treated with trimethoprim sulfamethoxazole, ciprofloxacin, and ampicillin. Carbapenems, nitrofutans, and cephalosporins of the 3rd and 4th generations are the most common antibiotics used to treat complicated UTIs (Foxman, 2010). The emergence of MDR- and XDR-UPEC strains has complicated the treatment of UTIs (Dehbanipour et al., 2016). In this study, MDR and XDR-UPEC strains from pediatric patients at HIMFG were analyzed for their phylogeny, integrons, and virulence profile. These strains were mainly associated with phylogenetic groups, B2 and D as described other studies; however, they also have been associated with phylogenetic group A (Ejrnæs et al., 2011; Zhao et al., 2015). These UPEC strains could be considered community strains with predisposing factors that facilitate UTIs in immunocompromised patients, as previously reported (Derakhshandeh et al., 2015; Rodrigues et al., 2015). UPEC strains of Mexican origin are mainly associated with phylogenetic group B2 (López-Banda et al., 2014). Moreover, ESBLs-producing MDR-UPEC strains were mainly associated with phylogenetic groups B2, A, and D, whereas the XDR-UPEC strains were associated with A, B2, and D. The recently reported distribution of UPEC strains associated with these phylogenetic groups agrees with our data (Zhao et al., 2015). Commensal *E. coli* strains with the ability to cause community-acquired UTIs have been related to phylogenetic group B1, which was not identified in this study (Mosquito et al., 2015). Remarkably, variations in phylogenetic groups are related to geographical area, infection site, and antibiotic resistance (Mokracka et al., 2011). Several adhesins and siderophores from UPEC strains participate in the colonization and persistence of bladder cells (Hannan et al., 2012). The genes that encode adhesins (EcpA, FimH, PapG, and CsgA) and the protein related to iron uptake (siderophores) were widely distributed in MDR- and XDR-UPEC strains associated with two phylogenetic groups (B2 and D) and to a lesser extent with A. Recently, UPEC strains associated with phylogenetic group B2 and D showed a high presence of siderophores, autotransporters protease, and adhesion genes, while a low presence of genes encoding toxins was also observed (López-Banda et al., 2014; Yahiaoui et al., 2015; Zhao et al., 2015). In addition, the *PapG*II variant gene from MDR- and XDR-UPEC strains associated with phylogenetic groups D and B2 was the most prevalent compared to the *papG*I and *papG*III variant genes. The *papG*II variant gene from UPEC strains associated with phylogenetic groups A and B2 is the most prevalent gene associated with cystitis in women and pyelonephritis in children and adults (Jantunen et al., 2000; Agarwal et al., 2013). However, four MDR-UPEC strains associated with phylogenetic group B2 and one strain associated with group D were associated with the *papG*III variant gene, which correlates with data reported by other authors (Agarwal et al., 2013).

The *bcsA* gene encoding cellulose is a component of the bacterial extracellular matrix in UPEC strains, and its expression is associated with the *csg* operon that codes for curli fimbriae (Saldaña et al., 2009). Our data showed the presence of the *bcsA* and *csgA* genes in MDR-UPEC strains associated with phylogenetic groups D, B2, and A. A study suggest that the expression of both structures is independent of the strain origin and participates in biofilm formation to protect against different antibiotics (Hung et al., 2013). A deletion in the *tosA* gene in CFT073 *E. coli* affected the colonization of the bladder and kidney in a murine model, indicating that TosA adhesin is required for the virulence of UPEC (Vigil et al., 2011a). The distribution of the *tosA* gene in MDR- and XDR-UPEC strains was associated with three main phylogenetic groups (A, B2, and D), which has been described in *E. coli* strains isolated from fecal matter, asymptomatic bacteriuria (ABU), cystitis, and pyelonephritis samples (Vigil et al., 2011b). Interestingly, a relationship between the presence of the *tos*A and *hly*A genes was identified in MDR-UPEC strains associated with phylogenetic group B2, while XDR-UPEC strains were related to D and B2. These associations have been described in UPEC strains collected from patients with pyelonephritis (Vigil et al., 2011b). The *tosA*-positive MDR- and XDR-UPEC strains included in this study showed a wide profile of virulence genes compared with *tosA*-negative strains and were associated with phylogenetic groups B2 and D. The *tosA* gene is considered a genetic marker of UPEC strains carrying several virulence genes (Vigil et al., 2011b).

Nosocomial pathogens containing integron classes are related to resistance to different antibiotic groups; however, this association in UPEC strains is poorly studied (Stalder et al., 2012). Class 1 integrons in hospital *E*. *coli* strains are the most frequently reported, followed by class 2 integrons (Poey and Laviña, 2014). A total of 24.2% of class 1 integrons in Mexican *E. coli* strains from different clinical sources were associated with MDR (Acosta-Pérez et al., 2015); similar data were obtained in our study. The highest presence of class 1 integrons was associated with phylogenetic groups D and B2 in MDR- and XDR-UPEC strains; however, several studies described variable results regarding the association of integrons and phylogenetic groups (Gündogdu et al., 2011; Zeighami et al., 2015). Sequencing the variable region of class 1 and 2 integrons from MDR- and XDR-UPEC strains revealed the presence of genes encoding the antibiotic modifying enzymes *aad*A1, *aad*B, *aac*C, *ant*1, *dfr*A1, and *dfr*A17. MDR- and XDR-UPEC strains with resistance to SXT and GM antibiotics were mainly associated with class 1 and 2 integrons, as described by other authors (Solberg et al., 2006; Márquez et al., 2008; El-Najjar et al., 2010; Soleimani et al., 2014; Acosta-Pérez et al., 2015; Yahiaoui et al., 2015). Class 3 integrons are part of the soil/freshwater proteobacteria group, have a poor occurrence rate and were not identified in this study (Deng et al., 2015).

Genetic diversity has been widely used to characterize populations of UPEC strains associated with several diseases of the urinary tract (Luo et al., 2012; Acosta-Pérez et al., 2015; Morales-Espinosa et al., 2016). The recurrence of UTIs at HIMFG was associated with several clones in the MDR-UPEC strains of phylogenetic groups D and B2, which are responsible for a majority of persistent UTIs. In contrast, the primary persistence of recurrent UTIs was associated with phylogenetic group B2, whereas clones associated with phylogenetic group D mainly caused reinfections by UPEC isolates (Luo et al., 2012). The persistence of UPEC strains is related to invasion, bacterial community, and quiescence, including the expression of several virulence genes (Hannan et al., 2012). Four clusters (II, III, IV, and V), two phylogenetic groups (B2 and D), class 1 integrons, and genes encoding fimbrial adhesin and iron uptake are associated with MDR- and XDR-UPEC strains. The *bcsA* gene and phylogenetic group D, were only related to the 557U clonal group of cluster II and to the 118U group of cluster V. These UPEC strains are associated with catheter-associated bacteremia and are thus likely to function in biofilm formation on the catheter using curli and cellulose (Hung et al., 2013). Clonal group 424U of cluster IV was related to the presence of the *iutD* and *hlyA* genes; thus, these genes could be located on the same pathogenicity island, as has been reported for PAI-*pheV*. Interestingly, cluster V presenting four clonal groups was characterized by the presence of the *tosA* gene and virulence genes, including *hlyA, bcsA, iutD*, and *papG*III. These data support the hypothesis that the TosA protein is a potential virulence marker in UPEC strains (Vigil et al., 2011a). Nonetheless, ESBL-producing clones associated with phylogenetic group B2 were only localized into the 532U clonal group of cluster III; likewise, the antibiotic resistance profiles showed that resistance to penicillin, beta-lactams, and cephalosporin was included in clonal group B2 (Mohajeri et al., 2014).

In conclusion, a high frequency of genes encoding virulence factors, a broad resistance profile associated with integrons (classes 1 and 2) and the ESBL phenotype are essential elements related to phylogenetic groups (B2 and D), which are distributed specifically in genetic clusters of MDR- and XDR-UPEC clinical strains. Additionally, these attributes confer to bacteria adaptive advantages to colonize, persist and facilitate UTIs caused by UPEC.

## Author contributions

Designed and conceived the experiments: SO and JX. Performed the experiments: SO, VL, FL, JR, and GE. Analyzed the data: SO, VL, AC, JA, MS, and JX. Contributed reagents/materials/analytical tools: SO, AC, VC, Dd, BL, IP, SG, RH, and JX. Wrote and reviewed the manuscript: SO, VL, AC, and JX.

## Funding

This work was supported by Public Federal Fund grants HIM/2013/007, HIM/2014/014, and HIM/2014/019 from the Hospital Infantil de México Federico Gómez.

### Conflict of interest statement

The authors declare that the research was conducted in the absence of any commercial or financial relationships that could be construed as a potential conflict of interest.
