# Supplementary material for: Multidrug- and Extensively Drug-Resistant Uropathogenic Escherichia coli Clinical Strains: Phylogenetic Groups Widely Associated with Integrons Maintain High Genetic Diversity
Source: Front Microbiol. 2016 Dec 21;7:2042. doi: 10.3389/fmicb.2016.02042 (PMC5174082; doi:10.3389/fmicb.2016.02042)
Supplement: Supplementary file 1 [file Table1.docx]

**Table S1**. Oligonucleotides specific to phylogenetic groups, virulence factors, resistance genes and amplification conditions for individual and multiplex-PCR assays.

| **Genes** | **Sequences 5’ – 3’** | Size in Base Pairs **(bp)** | **References** |
| --- | --- | --- | --- |
| *chuA* | ChuA-1: GACGAACCAACGGTCAGGAT  ChuA-2: TGCCGCCAGTACCAAAGACA | 279 | Clermont et al*.,* 2000 |
| *yjaA* | YjaA-1: TGAAGTGTCAGGAGACGCTG  YjaA-2: ATGGAGAATGCGTTCCTCAAC | 211 |  |
| *tspE*4.C2 | TspE4C2-1: GAGTAATGTCGGGGCATTCA  TspE4C2-2: CGCGCCAACAAAGTATTACG | 152 |  |
| *bscA* | bcsA-F: GAAGAATTCCTGACGCTGGCTAA  bcsA-R: TGAAAGCTTGGAACGCACTCATC | 2,767 | Saldaña et al*.,* 2009 |
| *ecpA* | ecpA-F: TGAAAAAAAAGGTTCTGGCAATAGC  ecpA-R: CGCTGATGAGGAGAAAGTGAA | 510 | Saldaña et al*.,* 2014 |
| *tosA* | TosA-F: GCACAGCATAACGGGAAAAT  TosA-R: CCAGCATGTTACCACGAATG | 589 | This study |
| *csgA* | CsgA-F: GGCGGATCCATGGACATGAAACTT  TTAAAAGTAGCAGC  CsgA-F: GCGAGCTCTTAGTACTGATGAGCGG  TCG | 185 | This study |
| *papG*I | PapGI-F: CAACCTGCTCTCAATCTTTACTG  PapGII-R: CATGGCTGGTTGTTCCTAAACAT | 692 | Tiba et al., 2008 |
| *papG*II | PapGII-F: GGAATGTGGTGATTACTCAAAGG  PapGII-R: TCCAGAGACTGTTCAAGAAGGAC | 562 |  |
| *papG*III | PapGIII-F: CATGGCTGGTTGTTCCTAAACAT  PapGIII-R: TCCAGAGACTGTGCAGAAGGAC | 421 |  |
| *fimH* | FimH-F: TGCAGAACGGATAAGCCGTGG  FimH-R: GCAGTCACCTGCCCTCCGGTA | 508 | Tiba et al*.,* 2008 |
| *iutD* | iutD-F: TACCGGATTGTCATATGCAGACCGT  iutD-R: AATATCTTCCTCCAGTCCGGAGAAG | 602 |  |
| *hlyA* | HlyA-F: CGTGGACACAGCTGCCAGCA  HlyA-R: TGCAGCGTGGCGGGCATCAT | 789 |  |
| *intI*1 | Int1-F: CAGTGGACATAAGCCTGTTC  Int1-R: CCCGAGGCATAGACTGTA | 160 | Gundogdu et al*.,* 2011 |
| *intI*2 | Int2-F: CACGGATATGCGACAAAAAGGT  Int2-R: GATGACAACGAGTGACGAAATG | 788 |  |
| *intI*3 | Int3-F: GCCTCCGGCAGCGACTTTCAG  Int3-R: ACGGATCTGCCAAACCTGACT | 979 |  |
| VR-int1 | Hep58: GTAGGGCTTATTATGCACGC  Hep59: CGGGATCCCGGACGGCATGCA | Variable product | Dillon et al., 2005 |
| VR-int2 | Hep74: CGGGATCCCGGACGGCATGC  ACGATTTGTA  Hep51: GATGCCATCGCAAGTACGAG | Variable product | Dillon et al*.,* 2005 |

*E. coli* haem-utilization-gene (*chuA*); uncharacterized protein YjaA from *E. coli* K12 (*yjaA*); anonymous fragment (*tspE*4.C2); cellulose: gene of subunit protein (*bscA*); *E. coli* common pilus (*ecpA*); type 1 secretion A (*tosA*); curli fimbriae (*csgA*); P fimbriae, PapG variant I (*papG*I); PapG variant II (*papG*II); PapG variant III (*papG*III); type 1 fimbriae (*fimH*); aerobactin (*iutD*); α-hemolysin (*hlyA*); class I integrase (*intl*1); class II integrase (*intl*2); class III integrase (*intl*3); variable region of class 1 integron (VR-int1); variable region of class 2 integron (VR-int2).

**Table S2.** Minimum inhibitory concentration (MIC) to 10 classes of antibiotics, phylogenetic groups, integron classes and production of ESBLs in 82 MDR-UPEC strains.

| **Clinical strains** | **Minimum Inhibitory Concentration to different classes of antibiotics (MIC, µg/mL)** | | | | | | | | | | | **PG** | **Class**  **Integron** | **ESBLs** | **Gender** | **Age**  **(Years)** |
| --- | --- | --- | --- | --- | --- | --- | --- | --- | --- | --- | --- | --- | --- | --- | --- | --- |
|  | **P** | **βL/I** | **C 1st-2nd** | **C 3rd** | **MB** | **FQ** | **CP** | **AG** | **FPI** | **TT** | **NF** |  |  |  |  |  |
| 118U1  118U2  118U3  118U4  118U5  502U1-0412 804U3-0412 | 128 | 4-8 | 2-8 | 0.5-1 | 1 | 0.5-2 | 0.5 | 8 | >256 | >256 | 4 | D  D  D  D  D  D  D | 1  1  1  1  1  1  1 | **-**  **-**  **-**  **-**  **-**  **-**  **-** | W  W  W  W  W  W  M | 17  17  17  17  17  5.0  6.0 |
| 1773U3 | >256 | 32-256 | 16-64 | 8 | 2 | 0.5-1 | 1 | 2 | >256 | >256 | 64 | D | **-** | **+** | W | 5.0 |
| 177U1  177U2  177U4  177U5  618U-0712 | >256 | 16-64 | 16 | 0.5-1 | 4 | 1-2 | 1 | 1 | >256 | >256 | 4 | D  D  D  D  D | -  -  -  -  - | -  -  -  -  - | W  W  W  W  W | 5.0  5.0  5.0  5.0  18 |
| 424U2 | >256 | 16-64 | 16 | 0.5-1 | 2 | 0.5-2 | 0.5-1 | 4 | >256 | >256 | 4 | D | - | - | W | 3.0 |
| 424U3  424U4  424U5 | >256 | 16-64 | 16 | 0.5-1 | 2 | 0.5-2 | 0.5-1 | 4 | >256 | >256 | 4 | D  D  D | -  -  - | -  -  - | W  W  W | 3.0  3.0  3.0 |
| 557U1  557U2  557U3  557U4  557U5 | >256 | 16-64 | 16 | 0.5-1 | 32 | 8 | 1 | 2 | >256 | >256 | 4 | D  D  D  D  D | -  -  -  -  - | -  -  -  -  - | M  M  M  M  M | 11  11  11  11  11 |
| 117U1-0512 | >256 | 16-128 | 2-8 | 0.5-1 | 4 | 1 | 8 | 0.5 | >256 | >256 | 2 | D | 1 | - | W | 1.5 |
| 173U5-0512 | 64 | 16-256 | 16 | 0.5-1 | 2 | 0.125-2 | 05 | 4 | 0.25 | 0.25 | 8 | D | - | - | W | 18 |
| 179U-1012 | >256 | 16-256 | 1-4 | 0.125-0.5 | 0.125 | 0.065-0.25 | 0.25 | 32 | >256 | >256 | 1 | D | 1 | - | W | 13 |
| 188U-1112 | >256 | 16-256 | 16 | 8 | 0.125 | 0.065-0.25 | 0.25 | 32 | >256 | 128 | 1 | D | 1 and 2 | - | W | 6.0 |
| 249U-1012 | >256 | 16-256 | 16 | 0.5-1 | 0.5 | 0.25-0.5 | 0.5-1 | >256 | >256 | >256 | 4 | D | - | - | M | 1.2 |
| 268U5-1012 | >256 | 16-256 | >256 | 8 | 2 | 256 | 0.5 | 1 | 0.5 | >256 | 1 | D | - | + | M | 5.0 |
| 310U5-0512 | >256 | 16-256 | 4-8 | 0.5-1 | 0.5 | 1 | 0.5 | 0.5 | >256 | >256 | 4 | D | 1 | - | W | 5.0 |
| 433U1-0512 | >256 | 16-256 | 16 | 0.25-1 | 0.5 | 2 | 0.5 | 0.5 | >256 | >256 | 4 | D | 1 | - | M | 0.1 |
| 446U-0912 | 64 | 16 | 16 | 0.25-1 | 4 | 0.125-0.5 | 1 | 0.5 | 0.25 | 16 | 4 | D | - | - | M | 5.0 |
| 494U2-0412 | >256 | 16-128 | 2-8 | 0.5-1 | 4 | 8-256 | 1 | >256 | 0.5 | 1 | 4 | D | 1 | - | W | 5.0 |
| 553U-1112 657U-0612 | >256 | 16-256 | 1-2 | 0.25-0.5 | 2 | 0.125-0.25 | 0.125 | 0.5 | >256 | >256 | 2 | D  D | 1  1 | -  - | W  W | ND  13 |
| 562U-0912 | >256 | 16-256 | 2-8 | 0.5-1 | 0.125 | 0.125-0.5 | 1 | >256 | >256 | >256 | 2 | D | - | - | W | 1.0 |
| 57U-1112 | >256 | 16-256 | >256 | >256 | >256 | 256 | 0.5 | >256 | 0.125 | 8 | 2 | D | - | + | M | 0.8 |
| 592U4-0412 | 16 | 16 | 16 | 0.25-1 | 0.125 | 0.125-0.5 | 0.5 | 0.25 | 0.5 | >256 | 4 | D | - | - | W | 0.3 |
| 606U1-0412 | 16 | 16-32 | 4-8 | 0.5-1 | 4 | 256 | 1 | 1 | 2 | >256 | >256 | D | 1 | - | W | 14 |
| 618U2-0412 | 128 | 16 | 4-8 | 0.25-1 | 0.5 | 16-32 | 0.5 | 16 | 0.5 | 4 | 8 | D | 1 | - | W | 16 |
| 86U-0612 | >256 | 16-256 | 8 | 0.5-1 | 0.5 | 1-2 | 0.5 | >256 | >256 | 4 | 8 | D | 1 | - | M | 6.0 |
| 909U-0612 | 4 | 16-128 | 64 | >256 | 1 | 8-256 | 2 | 2 | 0.25 | 8 | 4 | D | 1 | + | W | 1.3 |
| 992U-0912 | 64 | 16 | 0.5-1 | 0.5-1 | 0.5 | 0.25-2 | 8 | 8 | 0.25 | 0.25 | 4 | D | 1 | - | W | 2.0 |
| 42U-0612 | >256 | 16-128 | 256 | 256 | >256 | 0.125-2 | 256 | 0.5 | 0.25 | 0.25 | 4 | D | - | + | M | ND |
| 440U1 | >256 | 16-64 | 16-32 | 2 | 2 | 0.5-2 | 1 | 2 | >256 | 8 | 4 | B2 | 1 | + | W | 6.0 |
| 440U3 | >256 | 16-64 | 16-32 | 0.5-1 | 1 | 0.25-2 | 0.5-1 | 2 | >256 | 4 | 128 | B2 | 1 | - | W | 6.0 |
| 440U2  440U4  440U5 | >256 | 16-64 | 16-32 | 1 | 2 | 0.25-1 | 1 | 2 | >256 | 4 | 4 | B2  B2  B2 | 1  1  1 | -  -  - | W  W  W | 6.0  6.0  6.0 |
| 511U1  511U3  511U4  511U5 | >256 | 16-64 | 16-32 | 1 | 2 | 1-2 | 1 | 16 | >256 | >256 | 4 | B2  B2  B2  B2 | -  -  -  - | -  -  -  - | W  W  W  W | 6.0  6.0  6.0  6.0 |
| 511U2 | >256 | 16-64 | 16-32 | 1 | 2 | 0.5-2 | 2 | 16 | >256 | >256 | 8 | B2 | - | - | W | 6.0 |
| 21U-1112 | >256 | 16-128 | 16-64 | 0.5-1 | 0.125 | 0.25-2 | 0.5 | 16 | >256 | >256 | 4 | B2 | - | - | W | 16 |
| 532U1  532U3  532U5 | >256 | 16-32 | 16-64 | 8 | 0.5 | 0.065-0.5 | 0.125 | 0.5 | 0.125 | 4 | 2 | B2  B2  B2 | -  -  - | +  +  + | W  W  W | 13  13  13 |
| 532U2  532U4 | >256 | 16-32 | 16-32 | 0.25-1 | 0.5 | 0.125-0.5 | 0.25 | 0.125 | 0.125 | 4 | 2 | B2  B2 | -  - | -  - | W  W | 13  13 |
| 561U1  561U2  561U3  561U4  561U5 | >256 | 16-256 | >256 | 8-16 | 8 | 0.5-2 | 1 | 4 | >256 | >256 | 4 | B2  B2  B2  B2  B2 | 1  1  1  1  1 | -  -  -  -  - | M  M  M  M  M | 2.0  2.0  2.0  2.0  2.0 |
| 143U-0612  268U1-4-1012 | >256 | 16-256 | 256 | 8-16 | 8 | 256 | 0.5 | 0.5 | 0.5 | 8-256 | 4 | B2  B2 | -  - | +  + | W  M | 7.0  5.0 |
| 294U4-0512  70U-0612 | >256 | 16-256 | 4-8 | 0.25-0.5 | 0.125 | 0.125-0.5 | 0.25 | 0.25 | >256 | >256 | 2 | B2  A | 1  - | -  - | W  W | 0.3  7.0 |
| 44U-0612 | >256 | 16-256 | >256 | 8 | 8 | 256 | 0.5 | 1 | 0.5 | >256 | 8 | B2 | - | + | W | 16 |
| 529U-0712 | 32 | 4-8 | 2-4 | 0.125-0.5 | 0.5 | 0.25-0.125 | 0.25 | 1 | 4 | 8 | 1 | B2 | - | - | W | ND |
| 54U-0612 | 4 | 16-32 | 4 | 0.5-1 | 1 | 0.125-0.5 | 1 | 16 | 0.25 | >256 | 4 | B2 | 1 | - | W | 8.0 |
| 609U-0712 643U-0712 | >256 | 16-256 | 16 | 0.25-0.5 | 0.125 | 0.065-0.5 | 0.25 | 0.125 | >256 | >256 | 2 | B2  A | 1  - | -  - | W  W | 6.0  0.6 |
| 647U1-0712 | 16 | 16-64 | 16 | 16-32 | >256 | 0.25-1 | 1 | 0.5 | 0.5 | >256 | 8 | B2 | - | + | W | 8.0 |
| 876U3-0412 | 32 | 64-256 | >256 | 16-32 | >256 | 256 | 1 | >256 | 0.5 | 1 | 4 | B2 | - | + | M | 0.5 |
| 672U1-0612  674U-0612 | >256 | 16-128 | 32-256 | 0.5-1 | 4 | 2 | 1 | 4 | 2 | >256 | 8 | B2  B2 | 1  2 | -  - | W  M | 3.0  9.0 |
| 100U3-0612 | >256 | >256 | >256 | 32-64 | >256 | 256 | 1 | 2 | 0.5 | >256 | 2 | A | - | + | W | 3.0 |
| 503U-1112 | >256 | 16-256 | 2-8 | 0.25-0.5 | 0.125 | 0.25-0.5 | 0.25 | 0.5 | 0.25 | >256 | 1 | A | 1 | - | W | 9.0 |
| 84U-0912 | 8 | 16-64 | 16 | 1 | 0.5 | 1-2 | 1 | 16 | 2 | >256 | 8 | A | 1 | - | W | 2.0 |
| 647U-0712 | >256 | 16->256 | 16 | 1 | 0.5 | 0.25-1 | 0.5 | 1 | >256 | >256 | **8** | D | 1 | - | W | 8.0 |
| 618U-0412 | >256 | 32->256 | 32 | 1 | 0.5 | 0.25-1 | 0.5 | 1 | >256 | >256 | **8** | D | - | - | W | 16 |

Penicillins [P: ampicillin (AM)]; β-Lactam/β-Lactamase Inhibitor Combinations [βL/I: amoxicillin-Clavulanate (AMC), ticarcillin-clavulanate (TIM), piperacillin-tazobactam (TZP)]; Cephems of 1st and 2nd generations [C 1st-2nd: cephalothin (CF), cefaclor (CEC); Cephems of 3rd generation [C 3rd: ceftriaxone (CRO), ceftazidime (CAZ)]; Monobactams [MB: aztreonam (ATM)]; Fluoroquinolones [FQ: norfloxacin (NOR), ofloxacin (OFX)]; Carbapenems [CP: meropenem (MEM), imipenem (IPM)]; Aminoglycosides [AG: gentamicin (GM)]; Folate Pathway Inhibitors [FPI: trimethoprim-sulfamethoxazole (SXT)]; Tetracyclines [TT: tetracycline (TE)]; Nitrofurans [NF: nitrofurantoin (F/M)]; phylogenetic groups (PG); extended-spectrum beta-Lactamases (ESBLs); men (M); women (W); no data (ND).

**Table S3.** Minimum inhibitory concentration (MIC) to 10 classes of antibiotics, phylogenetic groups, integron classes, and production of ESBL in 21 XDR-UPEC strains.

| **Clinical strains** | **Minimum Inhibitory Concentration of the Antibiotic (MIC, µg/mL)** | | | | | | | | | | | **PG** | **Class of Integron** | **ESBL** | **Gender** | **Age**  **(Years)** |
| --- | --- | --- | --- | --- | --- | --- | --- | --- | --- | --- | --- | --- | --- | --- | --- | --- |
|  | **P** | **βL/I** | **C 1st-2nd** | **C 3rd** | **MB** | **FQ** | **CP** | **AG** | **FPI** | **TT** | **NF** |  |  |  |  |  |
| 424U1 | >256 | 32-256 | 32-256 | 16-32 | 32 | 0.5-2 | 0.25-0.5 | 16 | >256 | >256 | 2 | D | - | - | W | 3.0 |
| 513U-0912 | >256 | 32-256 | 64-256 | 16-64 | >256 | 1-2 | 0.25-0.5 | >256 | >256 | >256 | 64 | D | 1 | - | W | ND |
| 702U1-0912 | >256 | 32-256 | 256 | 8 | 8 | 256 | 0.5 | >256 | >256 | >256 | 4 | D | - | + | M | 2.0 |
| 736U1-0412 | >256 | 32-256 | 256 | 16-64 | >256 | 256 | 0.5-1 | 0.5 | 4 | >256 | 8 | D | - | + | M | 0.1 |
| 870U-0612 | >256 | 32-256 | 256 | 8-32 | 8 | 256 | 0.5 | 0.5 | >256 | >256 | 8 | D | - | + | W | ND |
| 851U-0612 | >256 | 32->256 | 256 | 16-32 | 64 | 256 | 64 | >256 | 1 | 4 | 4 | D | 1 | + | M | 9.0 |
| 877U-1112 | >256 | 32->256 | >256 | 256 | 2 | 8-128 | 256 | >256 | 1 | >256 | 4 | D | 1 | + | W | 7.0 |
| 945U2-0412 | >256 | >256 | >256 | 256 | >256 | 8-16 | 256 | >256 | 4 | >256 | 4 | D | 1 | + | M | ND |
| 965U5-0412 | >256 | 32->256 | 256 | 256 | 8 | 1-2 | 256 | >256 | 4 | >256 | 256 | D | - | + | W | 9.0 |
| 11U-0912 | >256 | 32-256 | >256 | 8-16 | 8 | 256 | 1 | 64 | >256 | >256 | 4 | B2 | 1 and 2 | + | M | 1.0 |
| 319U-0812 | >256 | 32-256 | 256 | 8 | 64 | 256 | 0.5 | >256 | 0.5 | >256 | 1 | B2 | - | - | W | ND |
| 54U1-0512 | >256 | 64-256 | 256 | 16-32 | 64 | 256 | 2 | 1 | >256 | >256 | >256 | B2 | - | + | W | 8.0 |
| 63U2-0512 678U-0712 | >256 | 32-256 | 256 | 16-32 | 32 | 256 | 0.5 | 32 | 0.25 | >256 | 1 | B2  B2 | -  - | +  + | M  W | 5.0  9.0 |
| 711U-0612 | >256 | 64-256 | 256 | 8-16 | 16 | 256 | 0.5 | >256 | >256 | >256 | 4 | B2 | 1 | + | M | 1.0 |
| 720U-0712 | >256 | 64-256 | 256 | 32-128 | 32 | 256 | 0.25 | 8 | 4 | 0.25 | 2 | B2 | 1 | - | M | 14 |
| 722U-0612 | >256 | 32-256 | 256 | 8-16 | 8 | 256 | 256 | 1 | >256 | >256 | 4 | B2 | - | + | M | 6.0 |
| 816U-1112 | >256 | 32-256 | 256 | 8-32 | 8 | 256 | 0.25 | >256 | 0.25 | >256 | 2 | B2 | - | + | W | 5.0 |
| 955U-0912 | >256 | 64-256 | >256 | 256 | >256 | 8-128 | 256 | >256 | 2 | >256 | 4 | B2 | - | + | W | 5.0 |
| 756U1-0412 | >256 | 32-256 | 256 | 8-16 | 16 | 256 | 0.5 | >256 | 1 | >256 | 4 | A | 1 | + | M | 3.0 |
| 807U1-0412 | >256 | 32-256 | 256 | 8-32 | 32 | 256 | 1 | 2 | >256 | >256 | 256 | A | 1 and 2 | + | M | 1.1 |

Penicillins [P: ampicillin (AM)]; β-Lactam/β-Lactamase Inhibitor Combinations [βL/I: amoxicillin-Clavulanate (AMC), ticarcillin-clavulanate (TIM), piperacillin-tazobactam (TZP)]; Cephems of 1st and 2nd generation [C 1st-2nd: cephalothin (CF), cefaclor (CEC); Cephems of 3rd generation [C 3rd: ceftriaxone (CRO), ceftazidime (CAZ)]; Monobactams [MB: aztreonam (ATM)]; Fluoroquinolones [FQ: norfloxacin (NOR), ofloxacin (OFX)]; Carbapenems [CP: meropenem (MEM), imipenem (IPM)]; Aminoglycosides [AG: gentamicin (GM)]; Folate Pathway Inhibitors [FPI: trimethoprim-sulfamethoxazole (SXT)]; Tetracyclines [TT: tetracycline (TE)]; Nitrofurans [NF: nitrofurantoin (F/M)]; phylogenetic groups (PG); extended-spectrum beta-Lactamases (ESBLs); men (M); women (W); no data (ND).
